# Plasma lipids signify the progression of precancerous gastric lesions to gastric cancer: a prospective targeted lipidomics study

**DOI:** 10.7150/thno.74770

**Published:** 2022-06-06

**Authors:** Zong-Chao Liu, Wen-Hui Wu, Sha Huang, Zhong-Wu Li, Xue Li, Guang-Hou Shui, Sin Man Lam, Bo-Wen Li, Zhe-Xuan Li, Yang Zhang, Tong Zhou, Wei-Cheng You, Kai-Feng Pan, Wen-Qing Li

**Affiliations:** 1 Key Laboratory of Carcinogenesis and Translational Research (Ministry of Education/Beijing), Department of Cancer Epidemiology, Peking University Cancer Hospital & Institute, Beijing, 100142, China.; 2 State Key Laboratory of Molecular Developmental Biology, Institute of Genetics and Developmental Biology, Chinese Academy of Sciences, Beijing, 100101, China.; 3 LipidALL Technologies Company Limited, Changzhou, 213022, Jiangsu Province, China.

**Keywords:** Gastric cancer, Lipidomics, Precancerous gastric lesion, Biomarker

## Abstract

**Rationale:** Gastric cancer (GC) is preceded by a stepwise progression of precancerous gastric lesions. Distinguishing individuals with precancerous gastric lesions that have progression potential to GC is an important need. Perturbated lipid metabolism, particularly the dysregulation of *de novo* lipogenesis, is involved in gastric carcinogenesis. We conducted the first prospective lipidomics study exploring lipidomic signatures for the risk of gastric lesion progression and early GC.

**Methods:** Our two-stage study of targeted lipidomics enrolled 400 subjects from the National Upper Gastrointestinal Cancer Early Detection Program in China, including 200 subjects of GC and different gastric lesions in the discovery and validation stages. Of validation stage, 152 cases with gastric lesions were prospectively followed for the progression of gastric lesions for a median follow-up of 580 days (interquartile range 390-806 days). We examined the lipidomic signatures associated with the risk of advanced gastric lesions and their progression to GC. Our published tissue proteomic data were referred to further investigate highlighted lipids with their biologically related protein expression in gastric mucosa.

**Results:** We identified 11 plasma lipids significantly inversely associated with the risk of gastric lesion progression and GC occurrence. These lipids were integrated as latent profiles to identify 5 clusters of lipid expression that had distinct risk of gastric lesion progression. The latent profiles significantly improved the ability to predict the progression potential of gastric lesions (AUC: 0.82 vs 0.68, Delong's P = 4.6×10^-4^) and risk of early GC (AUC: 0.81 vs 0.55, P = 6.3×10^-5^). Significant associations were found between highlighted lipids, their biologically correlated proteins and the risk of GC, supporting the role of the pathways involving monocarboxylic acid metabolism and lipid transport and catabolic process in GC.

**Conclusions:** Our study revealed the lipidomic signatures associated with the risk of gastric lesion progression and GC occurrence, exhibiting translational implications for GC prevention.

## Introduction

Gastric cancer (GC) is one major public health threat with high morbidity and mortality worldwide 1. GC of the intestinal type predominates in high-risk geographic areas 2, and its occurrence experiences multistep cascade progression of gastric lesions, which evolve from superficial gastritis (SG), chronic atrophic gastritis (CAG), intestinal metaplasia (IM), and low-grade intraepithelial neoplasia (LGIN) to high-grade intraepithelial neoplasia (HGIN) and invasive GC 3,4. Studies of TCGA and other data have examined the molecular subtypes of GC, aiming to provide a roadmap for patient stratification and targeted therapies 5,6. However, while most GCs are diagnosed at locally advanced or advanced stages with unfavorable prognosis^7^, efforts are warranted to identify populations at particularly high-risk for progression of gastric lesions and development of GC, essential for improving the primary prevention and early detection of GC. Efficient biomarkers are therefore highly needed.

Lipids play essential roles in cellular functions related to the carcinogenesis process 8. Perturbated lipid metabolism, including increased lipid uptake, endogenous *de novo* fatty acid synthesis, fatty acid oxidation, and cholesterol accumulation, has been reported to promote tumor growth and progression 9-11. In addition, lipid content of phospholipids could compromise membrane fluidity and signal transduction which may in turn affect GC tumorigenesis and progression 12,13. In our recent study based on untargeted metabolomics covering carbohydrates, amino acids, nucleotides, polar lipids, and other metabolites; six lipids, including α-linolenic acid, linoleic acid, palmitic acid, arachidonic acid, sn-1 lysophosphatidylcholine (LysoPC)18:3, and sn-2 LysoPC20:3 stood out to have the most robust associations with the risk of early GC, with the first three also significantly associated with the risk of gastric lesion progression in a prospective analysis 14. These highlight the potential importance of the overall lipidomic profile underlying GC carcinogenesis 15. However, previous metabolomics studies of GC were restricted to water-soluble compounds and volatile metabolites 16, which lacked coverage and in-depth investigation for a wide range of lipids with potentially pivotal functions, thus leaving a knowledge gap on the full spectrum of lipidomic signatures associated with the development of GC.

Based on a total of 400 subjects from Linqu county, a well-recognized high-risk area in eastern China 4,17, we conducted the first comprehensive lipidomics study for GC and delineated a plasma lipidomics profile for a sequence of gastric lesions and GC in two stages. We took advantage of our prospectively followed participants and longitudinally investigated the lipidomic signatures underlying the progression of gastric lesions and development of GC.

## Methods

### Study participants

Our study involved a total of 400 subjects in two stages from Linqu County, Shandong Province of China, an established high-risk area for GC, where most GCs are of the intestinal type 4,17. All subjects were enrolled from those attending the National Upper Gastrointestinal Cancer Early Detection (UGCED) Program for rural areas, in which residents aged 40 to 69 years received upper gastroendoscopy examinations free of charge. Individuals with cardiovascular, liver and spleen disorder and other major chronic diseases are ineligible for gastroendoscopy and were therefore excluded from the program. Gastroendoscopy was performed by two experienced gastroenterologists using video endoscopes (Olympus). For each individual, biopsies were taken at five standardized sites and other sites with suspicious lesion detected by endoscopy, if any 18. Formalin-fixed, paraffin-embedded tissue samples for biopsy were reviewed blindly by two pathologists. Each subject was given a global diagnosis of normal, SG, CAG, IM, LGIN, HGIN, or invasive GC, defined as the most severe gastric histology among all biopsies, following the criteria of the Updated Sydney System 18 and the Chinese Association of Gastric Cancer 19. Subjects were surveyed using standard questionnaires and had a 5ml blood sample collected following standardized collection process. H.pylori infection status was determined by enzyme-linked immunosorbent assay for plasma IgG 20.

**Figure 1:**
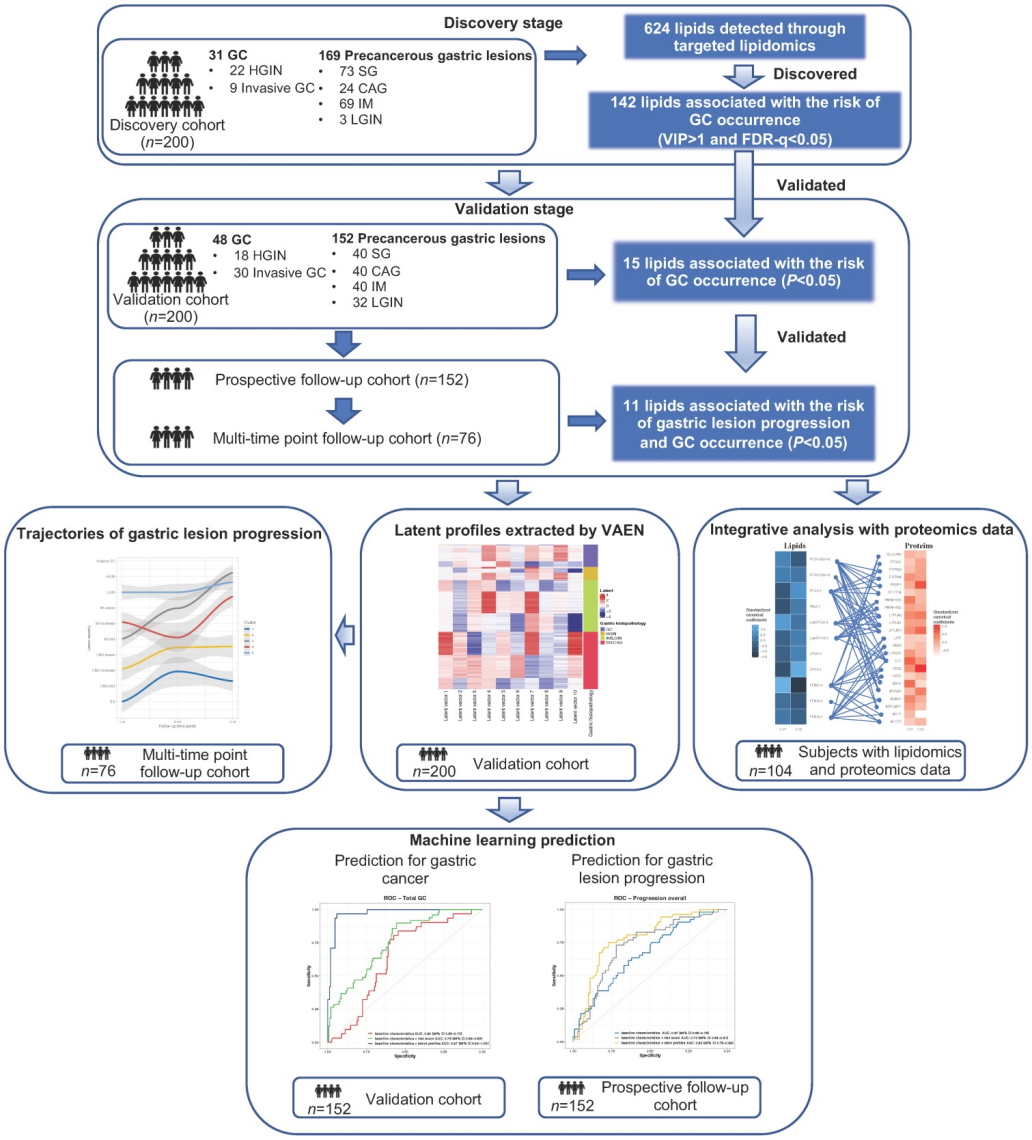
** General workflow of the study.** Targeted lipidomics analysis involved a total of 200 subjects in two stages respectively. In the validation stage, 152 non-GC subjects were prospectively followed for the progression of gastric lesions (“prospective follow-up cohort”). For 11 validated lipids significantly associated with risk of gastric lesion progression and GC occurrence, latent profiles were extracted using VAEN, representing the refined molecular pattern of lipids. Latent profiles of lipids were used to define lipidomic-based clusters of the prospective cohort subjects and the time-varying trajectories of gastric lesion progression were delineated by the clusters. XGBoost models were constructed to predict the risk of gastric lesion progression and GC occurrence. *CAG, chronic atrophic gastritis; FDR: false discovery rate; GC, gastric cancer; HGIN, high-grade intraepithelial neoplasia; IM, intestinal metaplasia; LGIN, low-grade intraepithelial neoplasia; ROC, receiver operating characteristic; SG, superficial gastritis; VAEN, variational auto-encoder followed by the elastic net regression model; VIP, variable importance in projection; XGBoost, extreme gradient boosting.*

The study consisted of two independent stages involving a total of 400 subjects. The discovery set included a total of 200 subjects with gastric lesions of different stages (n = 169) and GC (n = 31, including 22 HGINs and 9 invasive GCs) diagnosed in 2018. The validation set further independently enrolled 200 subjects, including 48 cases of GC and 152 cases with different gastric lesions diagnosed in 2017. We did not include any subjects with normal gastric mucosa as few of the adult residents had completely normal histology 17,19. We prospectively followed the subjects of gastric lesions in the validation stage (n = 152, “prospective cohort”) until May 31, 2021, for a median follow-up of 580 days (interquartile range 390 to 806 days), with endoscopic examinations conducted at the endpoint for each individual. Among them, we had a multi-time point longitudinal sub-cohort of 76 participants who undertook further gastroendoscopy examinations in the middle of follow-up and thus had three or more measurement of gastric lesions during the follow-up. The progression of gastric lesions during the follow-up for the prospective cohort, or during a time window for the multi-time point longitudinal sub-cohort was assessed based on the global diagnosis of gastric lesions, defined as the most severe gastric histology among all biopsies (SG, CAG, IM, LGIN, HGIN or invasive GC). Subjects were considered to have progression of gastric lesions, if the severity of gastric lesion at follow-up endpoint is higher than that at baseline. Details of the participants in each cohort are presented in **Figure 1** and Table S**1**.

The study was approved by the Institutional Review Board of Peking University Cancer Hospital. Informed consent was waived as study subjects were selected within the framework of the National UGCED Program.

### Targeted lipidomics profiling

Targeted lipidomics profiling was performed on plasma samples using ultra high-performance liquid chromatography-mass spectrometry (LC-MS) 21. Methods on sample preparation and LC-MS assays are detailed in the **Supplementary Methods**. Quality control (QC) samples were prepared using mixed plasma samples, with 1 QC sample inserted between every 20 tested samples. A total of 10 and 11 QC samples were inserted during the lipidomics profiling for plasma samples in the discovery and validation stage, respectively. Ionization signals were monitored in QC samples based on the intensities of internal standards for individual lipid classes to ensure no significant drop in intensity (within 20%) and no drift in retention time (within 0.05 min) throughout the run. Lipids were identified based on structure-specific multiple reaction monitoring (MRMs), which comprise MRMs specific to both head groups distinct to individual lipid classes and fatty acyl compositions, as well as correct retention times by comparing to authentic lipid reference compounds from human lipid ID inventory constructed in-house. Lipid levels were expressed in moles per L (mol/L) of plasma for statistical analyses.

### Bioinformatics and statistical analysis

We conducted bioinformatics and statistical analyses for the lipid signatures associated with the risk of GC compared with the well-recognized mild gastric lesion group (SG/CAG) or advanced gastric lesion group (IM/LGIN) as references, based on the discovery and validation set data, and the risk of gastric lesion progression based on the prospective cohort.

#### Identification and validation of key individual lipids

Data on lipid levels were log-transformed and normalized for analysis. Based on the discovery set data, Orthogonal Projections to Latent Structures Discriminant Analysis (OPLS-DA) was performed to calculate the variable importance projection (VIP) value between different comparison groups. Among lipids with VIP > 1 from OPLS-DA for the comparisons of GC with mild (SG/CAG) or advanced gastric lesion group (IM/LGIN), we used logistic regression models to calculate the odds ratios (ORs) and 95% confidence intervals (CIs) for their associations with GC respectively, adjusting for age, sex, and *H. pylori* infection. Lipids that had significant association (P-value < 0.05 and false discovery rate (FDR)-q value < 0.05) with GC, compared with mild or advanced gastric lesions, were examined during the validation stage, using logistic regression models adjusting for age, sex, and *H. pylori* infection. For the validated lipids (P < 0.05 in validation), meta-analysis was conducted for the associations with GC combining the discovery and validation sets. Validated lipids significantly associated with the risk of GC were further investigated for their associations with the progression of gastric lesions, based on the prospective cohort subjects. For this association analysis, the progression of gastric lesions for each subject was classified into three categories (regression, no-change, and progression), and ordinal logistic regression analyses were conducted, with P < 0.05 considered statistically significant. A Pearson's correlation coefficient matrix was derived to examine the pairwise correlation structure between the validated lipids, and pathway enrichment analysis was conducted on the validated lipids using MetaboAnalyst (https://www.metaboanalyst.ca/).

#### Applying generative neural networks to refine the latent profiles of key lipids

Focusing on the key lipids associated with the risk of GC and gastric lesion progression, we applied the variational autoencoder (VAE) framework, an unsupervised deep neural network, to decipher the non-linear nature of biological connections of lipid alterations with the risk of GC and progression of gastric lesions based on validation set subjects 22. The VAE model followed by the Elastic Net (EN) method, namely the VAEN strategy, was employed to extract the latent profiles (i.e., a latent matrix) that contained denoised information of the original lipid data 23. We generated 200 latent matrices from VAE models and fitted EN regression models (α = 0.5) on each matrix with 5-fold cross-validation. The predictive latent vector dimensions selected from each EN model were then evaluated by average *R*^2^ from a standard multivariate linear regression via 10-fold cross-validation. Latent matrix with the highest average *R*^2^, which indicated the best model efficiency, was kept for further analyses. Details are shown in the **Supplementary Material**.

#### Visualizing the changing trajectories of gastric lesions by clustering latent profiles of lipids expression

Taking advantage of the longitudinal follow-up of subjects with gastric lesions as one clear feature of the study design, we sought to further decipher whether individuals' patterns of gastric lesion progression would differ by the clusters of latent profiles of key lipids. The Partitioning Around Medoids (PAM) clustering method was used to derive the clusters for each individual of the prospective cohort (n = 152) 24. The optimal number of clusters was determined by Silhouette's method 25. We then examined the associations between the clusters of latent profiles and risk of gastric lesion progression, utilizing data from the prospective cohort. For each cluster, a time-variate trajectory depicting participants' average changes of lesion severity was plotted via the generalized additive model. The ORs (95% CIs) for the clusters associated with gastric lesion progression versus non-progression were calculated using the logistic regression model, adjusting for age, sex, *H.pylori* infection, and baseline gastric histopathology.

### Constructing machine learning risk prediction models for the risk of GC and gastric lesion progression

Machine learning models were trained upon the discovery set and tested on the validation set to evaluate the efficacy of the validated lipids as potential biomarkers. A multi-class XGBoost model was used to evaluate the efficacy of latent profiles in discriminating four case groups of mild gastric lesions, advanced gastric lesions, HGIN and invasive GC. Binary XGBoost models were further constructed for the risk prediction of total GC, HGIN, and invasive GC based on the validation set, and the risk prediction of gastric lesion progression based on the prospective cohort. For each outcome of interest, we developed a base model only including baseline characteristics such as age, sex, *H.pylori* infection, and baseline gastric histopathology (for the prediction of gastric lesion progression), as well as an updated model additionally integrating aforementioned latent profiles of key lipids. We also sought to integrate the risk scores of several or all 11 lipids with the base model, with risk scores calculated as the linear combination of the individual lipid levels and their coefficient estimates of logistic regression. For all prediction models, the prediction error was estimated by 10-fold cross-validation 26. Receiver operating characteristic (ROC) curves were plotted, with area under the curve (AUC) calculated. In addition, the Micro-average AUC was calculated to display the overall performance of the multi-class model 27. Delong test was used to compare the performance of prediction models with and without integrating the lipidomic signatures.

### Integrative analysis of the lipidomic and proteomic profiling

To provide clues for the biological mechanisms underlying the validated lipids associated with GC development, we referred to our recent published proteomics profiling results 28 and explored their potential correlations with the validated lipids in the current study. Combining with our proteomics data, we have 104 subjects available for both plasma lipidomics and tissue proteomics profiling data in the current study. The highlighted lipids in our study were then matched with their biologically related protein expression in gastric mucosa according to the annotation of Human Metabolite Database (HMDB). We assessed the overall correlation between plasma lipid levels and matched protein expression using the Wilks' λ test in the canonical correlation analysis (CCA) 29, a typical method to represent the correlation between two separate datasets. Significant canonical covariates (CVs) were identified based on the Hotelling-Lawley Trace (HLT) test and Pearson correlation analysis. Standardized canonical coefficients were calculated for visualizing the associations of each individual protein with the selected CVs. Pathway enrichment analyses were conducted for proteins significantly associated with the risk of GC.

## Results

### Key individual lipids associated with the progression of precancerous gastric lesions to gastric cancer

Characteristics of 400 study subjects are shown in **Table S1**. Principle component analysis showed that the QC samples were highly correlated, with the Spearman correlation coefficients (r) ranging from 0.96 to 1 (**Figure S1A-1D**), indicating high stability and reproducibility. QC samples showed good consistency with tested samples in quantification of plasma lipid levels (**Figure S1E-S1F**).

**Figure 2:**
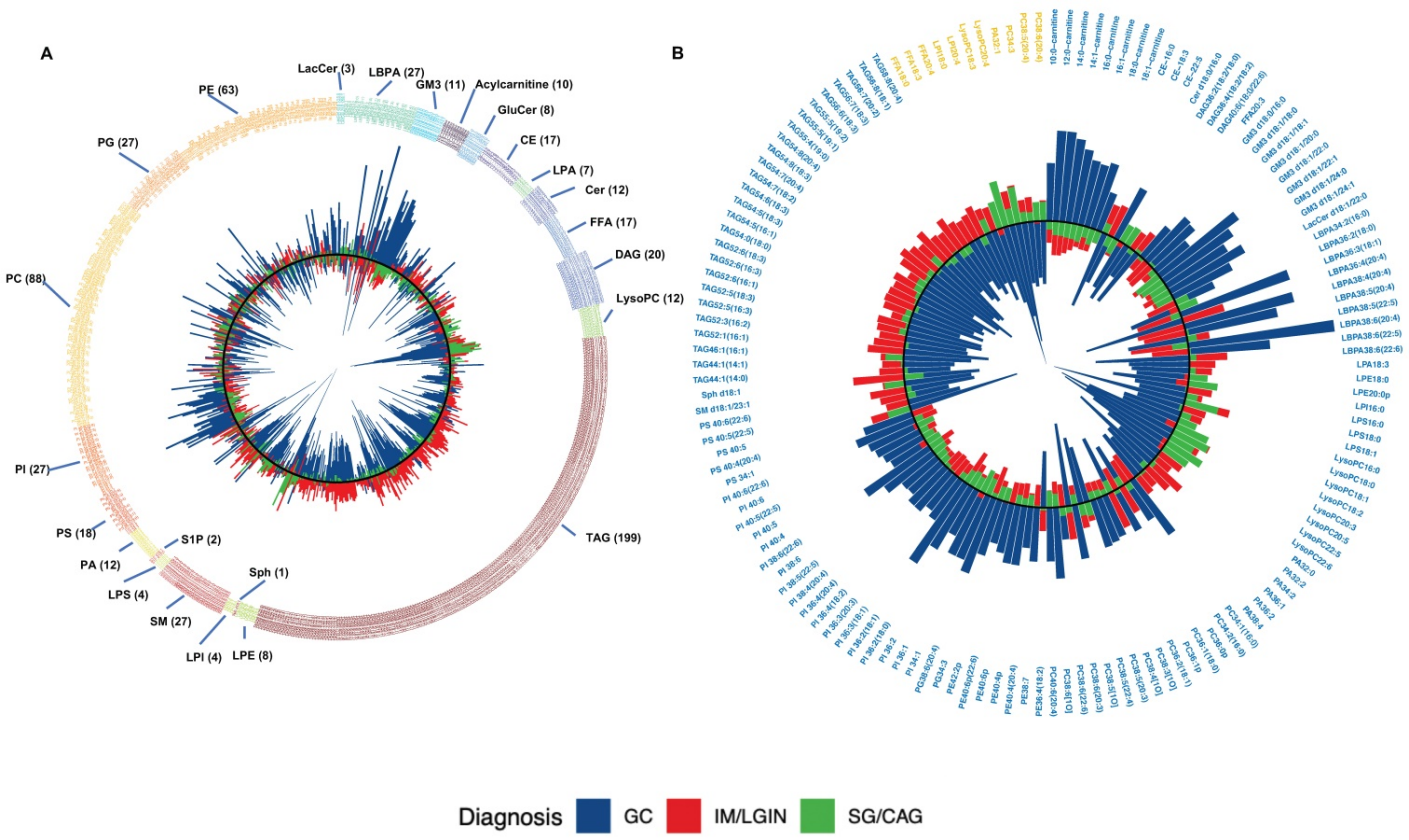
** Identification of the lipids through targeted lipidomics analyses in the discovery and validation stage. A.** A total of 624 lipids identified in the discovery stage. Lipid classes are displayed by different colors. **B.** Average levels of the 142 lipids associated with the risk of GC (VIP > 1 and FDR-q < 0.05) in the discovery stage. VIP values were calculated by orthogonal projections to latent structures discriminant analysis. Logistic regression adjusting for sex, age, and *Helicobacter pylori* infection was used for the association analyses. Of 142 lipids, 11 lipids associated with the risk of gastric lesion progression and GC occurrence are yellow-colored. For both panels, average lipid levels are shown for subjects with mild (green bar), advanced gastric lesions (red bar) and GC (blue bar). The inner-circle in black color is a reference line for lipid level equal to 0 and height of the bar indicates the lipid levels with log-transformation and normalization. The direction of bars pointing towards the center represents a lower lipid level and the direction pointing away from the center represents an increased lipid level in a subject group. *CAG, chronic atrophic gastritis; CE, cholesterol ester; Cer, ceramide; DAG, diacylglycerol; FFA, free fatty acid; FDR: False discovery rate; GC, gastric cancer; GluCer, glucosylceramide; GM3, monosialodihexosylganglioside; IM, intestinal metaplasia; LacCer, lactosylceramide; LBPA, lysobisphosphatidic acid; LGIN, low-grade intraepithelial neoplasia; LPA, lysophosphatidic acid; LPE, lysophosphatidylethanolamine; LPI, lysophosphatidylinositol; LPS, lipopolysaccharides; LysoPC, lysophosphatidylcholine; PA, phosphatidic acid; PC, phosphatidylcholine; PE, phosphatidylethanolamine; PG, glycerophospholipid; PI, phosphatidylinositol; PS, phosphatidylserine; SG, superficial gastritis; SM, sphingomyelin; Sph, sphingosine; S1P, sphingosine-1-phosphate; TAG, triacylglycerol; VIP, variable importance in projection.*

**Figure 3:**
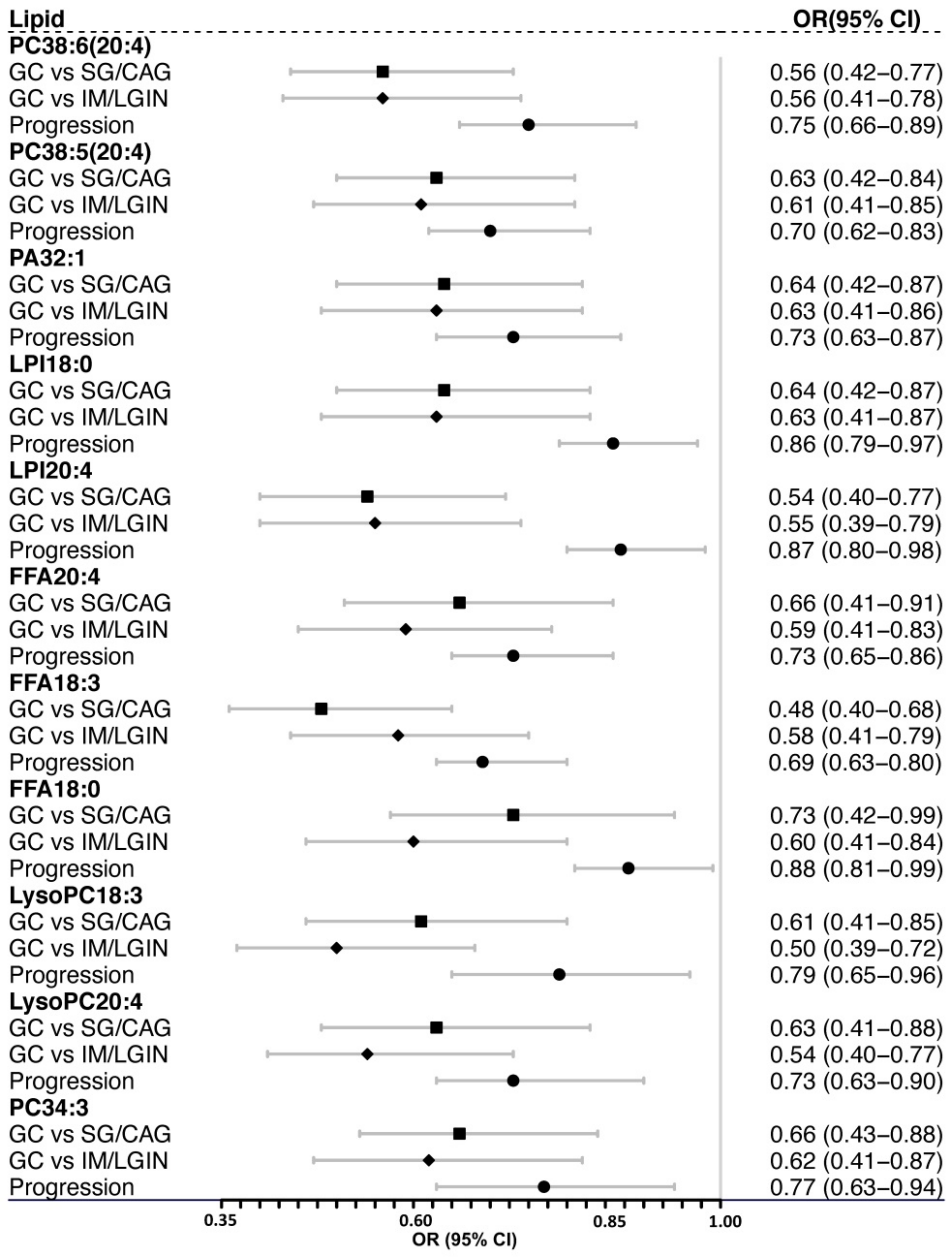
** The ORs (95% CIs) for the validated lipids associated with the risk of gastric lesion progression and GC occurrence.** ORs (95% CIs) for GC risk were calculated by logistic regression adjusting for age, sex, and *Helicobacter pylori* infection, combining the discovery and validation stage subjects for meta-analysis. ORs (95% CIs) for the risk of gastric lesion progression were calculated by ordinal logistic regression adjusted for age, sex, *Helicobacter pylori* infection and gastric histopathology, based on the prospective cohort. *CAG, chronic atrophic gastritis; CI, confidence interval. FFA, free fatty acid; GC,* gastric cancer*; IM, intestinal metaplasia; LGIN, low-grade intraepithelial neoplasia; LPI, lysophosphatidylinositol; LysoPC, lysophosphatidylcholine; OR, odds ratio; PA, phosphatidic acid; PC, phosphatidylcholine; SG, superficial gastritis.*

We identified 624 lipids in the discovery stage, including 199 triacylglycerols (TAGs), 88 phosphatidylcholines (PCs), 63 phosphatidylethanolamines (PEs), 27 phosphoinositol (PIs), 27 Sphingomyelins (SMs), 27 Phosphatidylglycerols (PGs), 27 Lysobisphosphatidic acids (LBPAs), 20 Diacylglycerol (DAGs), and 146 others (**Figure 2A**). Of them, 178 lipids had distinct plasma levels in GC from mild (SG or CAG) or advanced gastric lesion (IM or LGIN) group (VIP > 1). Compared with subjects with mild or advanced gastric lesions as reference respectively, a total of 142 out of 178 lipids were further associated with the risk of GC in logistic regression analyses (FDR-q < 0.05) (**Figure 2B**). We then sought to validate the associations for these lipids using an independent validation set, where 15 lipids showed consistent associations with GC (P < 0.05). Further analysis based on the prospective cohort found that 11 lipids (3 FFAs and 8 phospholipids) were also inversely associated with the risk of gastric lesion progression (P < 0.05), including PC38:6(20:4), PC38:5(20:4), PC34:3, LysoPC18:3, LysoPC20:4, LPI18:0, LPI20:4, FFA20:4 (arachidonic acid), FFA18:3 (α-linolenic acid), FFA18:0 (stearic acid), and PA32:1 (Table 1). Most of these lipids showed positive pairwise correlations (**Figure S2**). The ORs (95% CIs) for these 11 lipids associated with GC in meta-analysis combining the discovery and validation sets are shown in **Figure 3**.

Of the highlighted lipids, FFA20:4 (arachidonic acid), FFA18:3 (α-Linolenic acid), and LysoPC18:3 were identified as key metabolites for GC, and α-Linolenic acid was further associated with risk of gastric lesion progression in our published study on untargeted metabolomics 14, with similar effect magnitudes for associations in previous and current studies. Although the association with FFA18:2 (linoleic acid), FFA16:0 (palmitic acid), and LysoPC20:3 was not statistically significant in the present study, the association went to the same direction with similar effect magnitude (**Table S2**).

Pathway enrichment analysis revealed that the pathways of arachidonic acid metabolism (impact = 0.36; P = 0.005), α-linolenic acid metabolism (impact = 0.25; P = 6.41×10^-4^), linoleic acid metabolism (impact = 0.25; P = 0.016), and glycerophospholipid metabolism (impact = 0.12; P = 0.005) were among the top enriched pathways associated with GC and gastric lesion progression (**Table S3**).

### Latent profiles of key individual lipids

**Figure 4:**
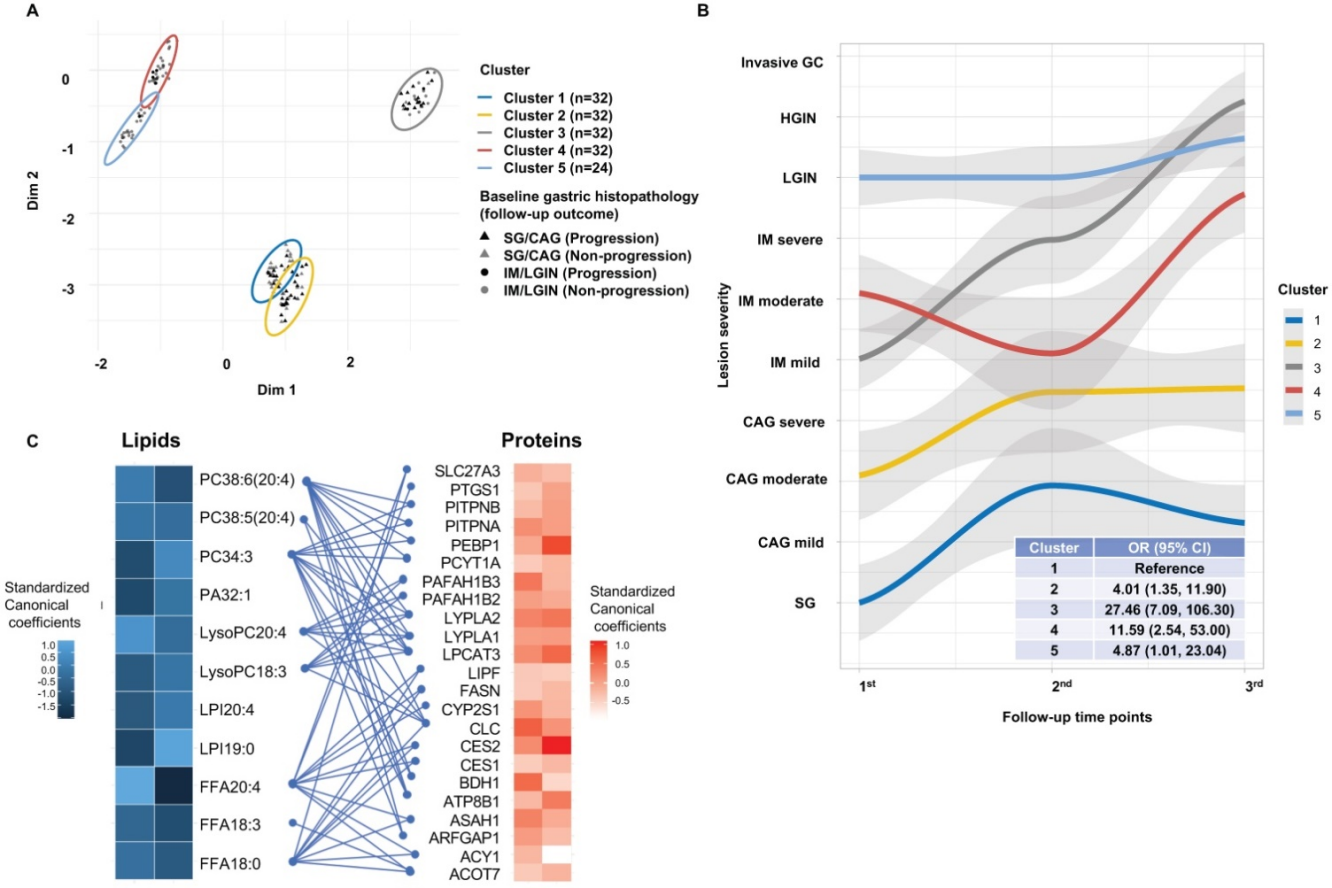
** Lipids latent profiles revealing clustered patterns of gastric lesion progression and the integrative analysis of the lipidomic and proteomic profiling. A.** Clusters of the individuals generated through the unsupervised PAM clustering method. The clusters are visualized within the first and second components derived from PCA. Five clusters are displayed with different colors. Baseline gastric histopathology are shown for subjects with SG/CAG (triangles) and IM/LGIN (circles). The black and grey color indicates whether a subject had or did not have gastric lesion progression, respectively. **B.** Time-varying trajectories depicting the average change of gastric lesion severity for each cluster. The ORs (95% CIs) for gastric lesion progression of each cluster were calculated by the logistic regression, using cluster-1 as the reference. **C.** Standardized canonical coefficients for the significant CVs in CCA. The standardized canonical coefficients for each CV are displayed in each cell with gradient color from black to blue for lipids and from white to red for proteins. The lipids are linked to their biologically relative proteins by blue edges. *CAG, chronic atrophic gastritis; CCA, canonical correlation analysis; CI, confidence interval; CV, canonical variate; FFA, free fatty acid; GC, gastric cancer; IM, intestinal metaplasia; LGIN, low-grade intraepithelial neoplasia*; *LPI, lysophosphatidylinositol; LysoPC, lysophosphatidylcholine; OR, odds ratio; PA, phosphatidic acid; PAM, partition around medoids; PC, phosphatidylcholine; PCA, principle component analysis; SG, superficial gastritis.*

Latent profiles of the 11 validated lipids were extracted by VAEN based on the validation set, where the resultant latent matrix was selected with an average *R^2^* = 0.90 (**Figure S3**). Applying PAM on the latent profiles, we defined 5 lipidomic-based clusters of the prospective cohort subjects. The clusters were visualized by principle component analysis (PCA) with different gastric histopathology and the progression of gastric lesions during follow-up (**Figure 4A**). Among subjects of the prospective cohort, the time-varying trajectories of gastric lesion progression were plotted to depict the changing lesion severity for each cluster, revealing diverse progression patterns with various start points of lesion severity (**Figure 4B**). Analysis of the changing trajectories of gastric lesions found that the risk of gastric lesion progression varied by clusters (F = 10.30, *P* = 2.3×10^-8^). Compared with cluster-1, the OR (95% CIs) for the risk of progression was 4.01 (1.35-11.90) for cluster-2, 27.46 (7.09-106.30) for cluster-3, 11.59 (2.54-53.00) for cluster-4, and 4.87 (1.01-23.04) for cluster-5.

### Integrative analysis of tissue proteomic and plasma lipidomic data

Through annotation by HMDB, we identified 179 proteins that were biologically related to the 11 key lipids, 23 proteins among which were then matched in our published proteomics database (**Table S4**). The CCA showed statistically significant correlations between the matched protein expression and key lipid levels (Wilks' λ test P = 0.001) with 2 significant CVs (CV1: Pearson's r = 0.75, HLT P = 0.001; CV2: Pearson's r = 0.68, HLT P = 0.04). The standardized canonical coefficients of individual proteins with CV1 or CV2 are shown in **Figure 4C**. Of the proteins, 5 FFA-related proteins (PTGS1, ASAH1, SLC27A3, CES2, ACY1) and 7 phospholipids-related proteins (PEBP1, LYPLA2, PITPNB, PITPNA, PAFAH1B2, ATP8B1, BDH1), were significantly associated with the risk of GC compared with mild or advanced gastric lesions (**Table S4**). These significant proteins were enriched in the gene ontology pathways involving monocarboxylic acid metabolism (P = 4.02×10^-4^), lipid transport (P = 0.005) and catabolic process (P = 0.023) associated with GC (**Figure S4**).

### Prediction models for the risk of GC and gastric lesion integrating lipidomic signatures

**Figure 5:**
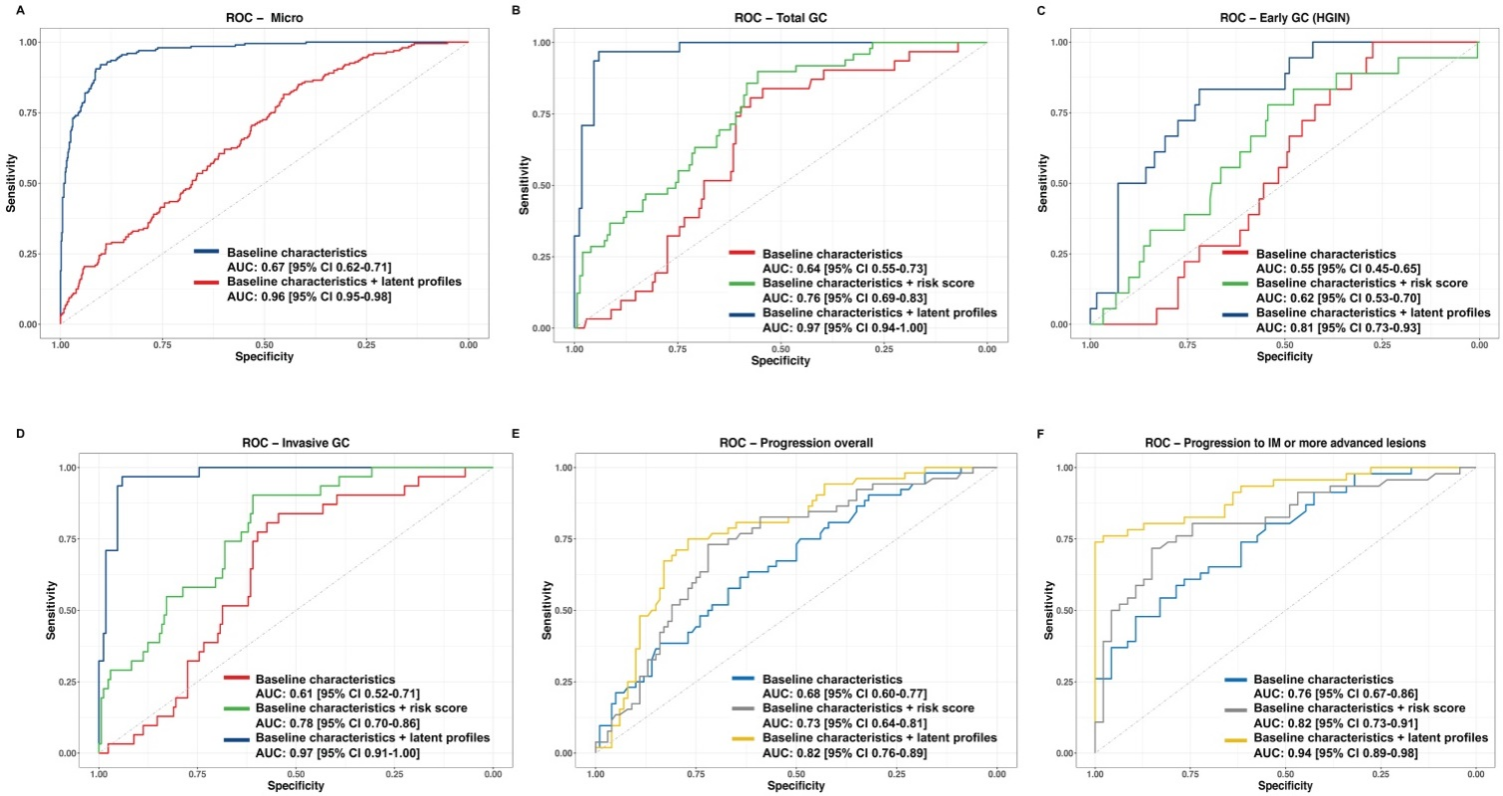
** Prediction models for the risk of gastric lesion progression and GC occurrence integrating lipid profiles. A.** Micro-average AUC displaying the overall performance of predicting gastric histopathology; **B.** AUC for predicting total GC (invasive GC + HGIN); **C.** AUC for predicting early GC (HGIN); **D.** AUC for predicting invasive GC; **E.** AUC for predicting overall gastric lesion progression; **F.** AUC for predicting individuals' progression to IM or more advanced lesions. For each outcome of interest, we developed a base model only including baseline characteristics, a model additionally integrating risk scores from individual lipid levels and a model additionally integrating lipid profiles. ROC curves were plotted by each model, and Delong's tests were used to compare AUC of the ROC curves for the two models. Specifically, the micro-average AUC was calculated for evaluating the multi-class prediction model. *AUC, area under the curve; CI, confidence interval; GC, gastric cancer; HGIN, high-grade intraepithelial neoplasia; IM, intestinal metaplasia; ROC, receiver operating characteristic.*

The trained XGBoost classifier was tested on the validation set. Compared with the model including only baseline characteristics, the model integrating the lipid latent profiles in the validation set showed significant improvement in the prediction on the overall gastric histopathology (AUC (95% CI): 0.96 (0.95-0.98) vs 0.67 (0.62-0.71), Delong's P < 0.001, **Figure 5A**), and the prediction on total GC (0.97 (0.94-1.00) vs 0.64 (0.55-0.73), P < 0.001, **Figure 5B**), either for invasive GC or early GC (**Figure 5C-5D**). Adding lipidomic signatures also yielded better prediction performance for the overall progression from any stage of gastric lesions (0.82 (0.76-0.89) vs 0.68 (0.60-0.77), Delong's P < 0.001, **Figure 5E**) and for the progression to IM or more advanced gastric lesion (0.94 (0.89-0.98) vs 0.76 (0.67-0.86), P < 0.001, **Figure 5F**). A forward stepwise strategy using logistic regression was adopted to derive the best combination of key lipids levels for risk score calculation, where combining all the 11 lipids finally showed the best performance compared with combining several of them. The performance of the prediction model integrating the latent profiles exhibited advantageous performance than the model integrating the risk score (**Figure 5B-5F**).

## Discussion

In our population-based targeted lipidomics study, we comprehensively revealed the lipidomic fingerprints associated with the progression of gastric lesions and risk of GC. Eleven key lipids were significantly associated with the risk of GC in both the discovery and validation stages, and were also inversely associated with the risk of gastric lesion progression in the prospective study, which was further corroborated by the analysis of the changing trajectories of gastric lesions during multi-time point endoscopic follow-up. These lipids were integrated as latent features to train XGBoost models, which significantly improved the ability to predict the progression potential of gastric lesions and risk of early GC. Integrative analyses were conducted utilizing our published proteomics data, which yielded significant associations between highlighted lipids, their biologically correlated proteins and the risk of GC, supporting the role of pathways involving monocarboxylic acid metabolism and lipid transport and catabolic process in GC.

Previous metabolomics studies based on tissues, blood and urine samples have examined lipid metabolites in GC, as summarized in our systematic review 30 and other recent studies 14,31. Despite limited coverage of lipids, often restricted by a modest sample size and lack of a validation stage, those studies provided evidence supporting possible lipid dysregulations, particularly the alterations of SMs, PCs, and PEs in GC, but consistent findings were sparse 30. Few studies have focused on the broad lipidomic profile, which represents a comprehensive collection of lipids within a biological system, associated with GC previously. *Lee et al.* compared the plasma lipid profile between 20 cases of GC and 20 non-cancer controls, which revealed alterations of PCs (PC34:2, 36:3, and 36:4) and LPA18:2 in GC 32. *Hung et al.* also conducted a small-scale study with 18 GC cases and reported distinct lipidomic profiles of GC from noncancerous tissues 13. Two studies have focused specifically on phospholipids associated with GC. One study only included 36 samples (20 GCs and 16 controls) 33. The other study enrolled 199 subjects with several different gastric lesions but the scope was limited, with only 54 phospholipids tested 34.

In our study, the 11 highlighted key lipids included 8 phospholipids and 3 FFAs. Except three lipids (FFA18:0, LPI18:0, and PA32:1), FFA18:3 (α-linolenic acid) and FFA20:4 (arachidonic acid) are polyunsaturated fatty acids (PUFAs), and other phospholipids contain PUFAs in chemical structure. FFA18:3 is an n-3 essential fatty acid mostly found in the chloroplast of green leafy vegetables, and FFA20:4 is an n-6 essential fatty acid usually found in meat, eggs and dairy products 35. These two PUFAs were covered in our recent untargeted metabolomics platform and significantly associated with GC risk 14. Reduction of PUFAs in tumor microenvironment has been reported to aid the escape of tumor cells from ferroptosis, an iron-dependent and non-apoptotic form of cell death associated with oxidized lipids 36. A recent study has shown that n-3 and n-6 PUFAs could selectively induced ferroptosis in cancer cells under ambient acidosis, and excess dietary intake of PUFAs might be a selective adjuvant antitumor modality 36. Although FFA18:0 does not belong to the group of PUFAs, it has been identified as a possible inhibitor of pyruvate dehydrogenase kinase, playing a pivotal role in metabolic reprogramming in cancers 37. In addition to the lipid-level association, integrative analysis of the proteomic data further supported the enriched fatty acid metabolism in GC development. For example, PTGS1, a FFA20:4-related protein, was positively associated with risk of GC and involved in the monocarboxylic metabolic process, the up-regulation of which may be stimulated by *H.pylori* infection, contributing to gastric prostaglandin E2 production, a pro-inflammatory eicosanoid in GC 38,39.

The newly unearthed phospholipids (3 PCs, 2 LysoPCs, 2 LPIs, and 1 PA) substantiated their potential importance for the progression of gastric lesions to early GC. Phospholipids are composed of two hydrophobic fatty acyl chains and one hydrophilic head group, varying by the chain length and degree of saturation of fatty acyl moieties. Foods with high phospholipid content include eggs, organ and lean meats, fish, shellfish, cereal grains and oilseeds 40. Phospholipids participate in lipid metabolism that provides biomass component for cancer cell proliferation and were shown to regulate the signaling molecules for uncontrolled cancer cell proliferation 41. An increase in PUFA-containing phospholipids was shown to contribute to the induction of ferroptosis in human cancer cells 42, coherent with the inverse association of these phospholipids associated with GC in our study.

PCs and LysoPCs are the major phospholipid subclasses with distinct levels between GC and non-neoplastic gastric lesions. PCs can be converted to LysoPCs via the cleaving action of phospholipase A_2_ or by the transfer of fatty acids to free cholesterol via lecithin-cholesterol acyltransferase 43. The downregulated polyunsaturated PCs in GC that we observed might be related to the suppressed biosynthesis of polyunsaturated lipids in tumor microenvironment activated by *de novo* lipogenesis 44. In addition, LysoPCs might be converted to lysophosphatidic acid that promotes cancer cell proliferation 45, leading to lowered LysoPC levels in cancer. Several key proteins biologically related to PCs and LysoPCs were significantly associated with GC risk, highlighting the potential importance of these phospholipids in gastric carcinogenesis. It is worth noting that a downregulated or absent PEBP1 expression has been associated with GC onset and its ability to invade and metastasize 46.

LPIs have been well-known to activate signaling cascades relevant to cancer cell proliferation and tumourigenesis 47. Although LPIs were found to be elevated in several types of cancers 47, findings on GC were sparse. The only one study that tested LPIs alterations reported prominently decreased level of the overall LPIs in GC 32, consistent with our findings. PA is the simplest phospholipid and can be found naturally in the vegetables, only in small quantities 48. The observed decreased level of PA32:1 in GC might be attributed to the increased phosphohydrolase activity of lipins, enzymes of the *de novo* lipogenesis pathway 49. Although our proteomics analyses did not cover the related proteins, recent data have demonstrated that lipin-1 may amplify the inflammatory process, thereby promoting carcinogenesis and tumor progression 50.

We sought to integrate the highlighted lipids for the prediction of gastric lesion progression and GC risk. We did not resort to a risk score-based model by directly integrating the regression coefficients of validated lipids given the strong collinearity of validated lipids, which might lead to biased estimates of the risk score for subgroup identification and risk prediction. Alternatively, we introduced the latent profile approach to extract the refined molecular pattern of lipids via generative neural networks, which has the advantage of capturing the complex non-linear relationship between multiple lipids and the research outcomes 51 and is appropriate for differentiating polytomous outcomes of interest with multi-classification 52.

Strengths of our study included a two-stage lipidomics study of different gastric lesions along the cascade of gastric carcinogenesis and GC, involving a total of 400 subjects with complete information on *H. pylori* infection and gastric histology for multivariate adjustment. We also had prospective follow-up of validation stage subjects, even with multi-time point endoscopic follow-up, allowing the longitudinal investigation of plasma lipids associated with the risk of gastric lesion progression to GC. This study has limitations. First, although we attempted to conduct a prospective study, only part of the subjects had multi-time point follow-up. The plasma lipidomic metabolites were measured only once at subject's enrollment, which reduced the likelihood of reverse causation but has precluded us from analyzing the time-varying lipids level with the evolution of gastric lesions. Second, all participants were enrolled from an area with high GC mortality and all samples were handled in a standardized manner. Notwithstanding the minimized residual confounding from host genetic background of subjects and ensured internal validity, our results might not be necessarily extrapolated to other low-risk populations. Third, the extrapolation of our findings should be cautious also considering that most GCs in Linqu county are of intestinal type, but the distribution of GC subtypes has clear geographical differences. External validation studies are needed to evaluate the lipidomic profiles of GC and replicate the highlighted individual lipids associated with GC in the current study. Fourth, despite a thorough targeted lipidomics study and the efforts of integrative analyses with proteomics data, our study cannot answer the underlying mechanisms for the observed associations. Fifth, our study was underpowered for evaluating the possible interactions or mediation effects of other GC risk factors on the associations with lipids. Sixth, plasma lipid profiles may not be fully representative of those in the gastric tissue, so findings from the integrative analyses with tissue proteomic data in our study should be interpreted with caution.

## Conclusions

In conclusion, our study revealed the lipidomic signatures may be associated with the risk of gastric lesion progression and GC occurrence, supporting the altered lipid metabolism in gastric carcinogenesis. Decreased plasma lipids show promise as noninvasive biomarkers for early detection of GC. The findings provide a solid reference for the primary intervention of GC and exhibit a translational value for precision medicine, aiming for early detection and management of GC. Future large-scale long-term prospective studies, particularly with repeated measurements of lipids level during the follow-up would be preferred for lipids validation before the translation of our findings into major public health strategies in large communities.

## Supplementary Material

Supplementary figures and tables.

## Figures and Tables

**Table 1:** Lipids Associated with the risk of GC and the progression of the gastric lesions

	Discovery cohort								Validation cohort				Prospective cohort	
	GC vs SG/CAG				GC vs IM/LGIN				GC vs SG/CAG		GC vs IM/LGIN		Progression vs Non-progression	
Lipid	OR	P value	VIP	FDR-q	OR	P value	VIP	FDR-q	OR	P value	OR	P value	OR	P value
PC38:6(20:4)	0.54	0.009	1.66	0.049	0.61	0.010	1.18	0.052	0.60	0.007	0.54	0.002	0.75	0.025
PC38:5(20:4)	0.62	0.007	1.39	0.039	0.74	0.037	0.94	0.103	0.64	0.013	0.54	0.003	0.70	0.021
PA32:1	0.51	0.003	1.59	0.024	0.50	0.005	1.49	0.036	0.78	0.108	0.71	0.047	0.73	0.022
LPI18:0	0.41	5.5×10^-4^	2.18	0.008	0.58	0.010	1.08	0.051	0.81	0.137	0.67	0.039	0.86	0.015
LPI20:4	0.13	1.2×10^-6^	3.56	1.9×10^-4^	0.13	4.1×10^-5^	2.71	0.003	0.72	0.047	0.67	0.024	0.87	0.014
FFA20:4	0.57	0.017	1.32	0.076	0.50	0.006	1.20	0.038	0.71	0.038	0.65	0.021	0.73	0.022
FFA18:3	0.37	6.7×10^-4^	2.14	0.009	0.50	0.006	1.39	0.040	0.56	0.004	0.63	0.015	0.69	0.025
FFA18:0	0.52	0.037	1.32	0.113	0.58	0.004	1.37	0.032	0.64	0.003	0.62	0.014	0.88	0.013
LysoPC18:3	0.23	3.7×10^-5^	3.06	0.001	0.31	8.3×10^-4^	2.05	0.012	0.81	0.139	0.61	0.018	0.79	0.049
LysoPC20:4	0.22	7.1×10^-6^	2.98	0.003	0.17	7.2×10^-5^	2.62	4.5×10^-4^	0.88	0.258	0.67	0.022	0.73	0.028
PC34:3	0.64	0.008	1.51	0.048	0.60	0.004	1.46	0.031	0.71	0.04	0.66	0.04	0.77	0.036

Abbreviations: CAG, chronic atrophic gastritis; GC, gastric cancer including high-grade intraepithelial neoplasia and invasive gastric cancer; IM, intestinal metaplasia; LGIN, low-grade intraepithelial neoplasia; SG, superficial gastritis; OR, odds ratio; VIP, variable importance in projection.

## Abbreviations

AUC: area under curve
CAG: chronic atrophic gastritis
CCA: canonical correlation analysis
CI: confidence interval
FDR: false discovery rate
FFA: free fatty acid
GC: gastric cancer
*H. pylori*: *Helicobacter pylori*
HGIN: high-grade intraepithelial neoplasia
IM: intestinal metaplasia
LC-MS/MS: liquid chromatography-tandem mass spectrometry
LGIN: low-grade intraepithelial neoplasia
LPI: lysophosphoinositol
LysoPC: lysophosphatidylcholine
GAM: general additive model
OPLS-DA: orthogonal projections to latent structures discriminant analysis
OR: odds ratio
PA: phosphatidic acid
PC: phosphatidylcholine
PCA: principal component analysis
QC: quality control
ROC: receiver operating characteristic
SG: superficial gastritis
UGCED: Upper Gastrointestinal Cancer Early Detection
VAE: variational auto-encoder
EN: elastic net
VAEN: variational auto-encoder followed by the elastic net regression
VIP: variable importance projection
XGBoost: extreme gradient boosting
