# Supplementary material for: Plasma lipids signify the progression of precancerous gastric lesions to gastric cancer: a prospective targeted lipidomics study
Source: Theranostics. 2022 Jun 6;12(10):4671–83. doi: 10.7150/thno.74770 (PMC9254242; doi:10.7150/thno.74770)
Supplement: Supplementary file 1 — Supplementary figures and tables. [file thnov12p4671s1.pdf]

Plasma lipids signify the progression of precancerous gastric lesions to gastric cancer: a prospective targeted lipidomics study

Supplementary Materials

Content

Supplementary methods

Figure S1 Quality control (QC) of the targeted lipidomics analysis

Figure S2 Pearson correlation matrix for the validated lipids

Figure S3 Latent profiles revealing specific patterns of lipid alterations behind GC and gastric lesion progression

Figure S4 Pathway analysis on the proteins significantly associated with the risk of GC

Table S1 Characteristics of the subjects in each study cohort

Table S2 Lipids associated with the risk of GC shown in the untargeted metabolomics and targeted lipidomics analysis

Table S3 Pathway analysis on the validated lipids

Table S4 The matched proteins associated with the validated lipids

Supplementary methods

Targeted lipidomics profiling

Sample preparation

Plasma (20 µL) for lipidomics analyses was inactivated via the addition of 750 µL of ice-cold chloroform: methanol (1:2) (v/v). Samples were vortexed for 15 s and then incubated for 1 h at 1500 rpm at 4 °C. At the end of incubation, 250 µL of ice-cold chloroform and 350 µL of ice-cold MilliQ water were added. Samples were vortexed for 15 s and put on ice for 1 min. This step was repeated once. Samples were then centrifuged at 12 000 rpm for 5 min 4 °C to induce phase separation. The lower organic phase was first extracted to a new tube. Then, another 450 µL of ice-cold chloroform was added to the remaining aqueous/methanol phase. Samples were vortexed briefly for 15 s and put on ice for 1 min, and centrifuged at 12 000 rpm for 5 min 4 °C. The lower organic phase was extracted and pooled together with the first round organic extract. Double rounds of extraction ensured a better recovery and reduce variations across samples. The remaining aqueous/methanol phase was then centrifuged at 12 000 rpm for 5 min 4 °C, and clean supernatant containing polar metabolites were extracted and transferred to new tube. The organic phase was dried in the SpeedVac under OH mode, while aqueous phase was dried under H2O mode. QC samples were prepared using mixed plasma samples according to identical steps as the actual plasma samples as aforementioned.

Liquid chromatography–mass spectrometry (LC-MS) detection

Electrospray ionization parameters were set as follows, curtain gas 20 psi, collision gas at high ion spray voltage at -4500 V, GS1 and GS2 were both at 25 psi, and source temperature was set at 400 °C. Lipid extracts were resuspended in 100 µL of chloroform: methanol (1:1) and spiked with equal volume of internal standard cocktail tailored for plasma lipid composition prior to LC-MS analysis [1]. Polar lipids were separated on a Phenomenex Luna Silica 3 µm column (i.d.

150x2.0 mm) under the following chromatographic conditions: mobile phase A (chloroform:methanol:ammonium hydroxide, 89.5:10:0.5) and mobile phase B (chloroform:methanol: ammonium hydroxide: water, 55:39:0.5:5.5) at a flow rate of 270 μL/min and column oven temperature at 25 ^{o}C. The gradient started with 5% of B and was held for 3 min, which was then increased to 40% of B over 9 min, and was held at 40% for 4 min before further increasing to 70% B over 5 min. The gradient was maintained at 70% B for 15 min before returning to 5% B over 3 min, and was finally equilibrated for 6 min. Glycerol lipids including diacylglycerols (DAG) and triacylglycerols (TAG) were quantified using a modified version of reverse phase HPLC/MRM [2]. Separation of neutral lipids were achieved on a Phenomenex Kinetex-C18 2.6 μm column (i.d. 4.6x100 mm) using an isocratic mobile phase containing chloroform:methanol:0.1 M ammonium acetate 100:100:4 (v/v/v) at a flow rate of 170 μL for 17 min. Levels of short-, medium-, and long-chain TAGs were calculated by referencing to spiked internal standards of TAG(14:0)3-d5, TAG(16:0)3-d5 and TAG(18:0)3-d5 obtained from CDN isotopes, respectively. DAGs were quantified using d5-DAG16:0/16:0 and d5-DAG18:1/18:1 as internal standards (Avanti Polar Lipids). Free cholesterols and cholesteryl esters were analysed as described previously with d6-cholesterol and d6-C18:0 cholesteryl ester (CE) (CDN isotopes) as internal standards [3]. Samples were randomized on the LC/MS queue, with 1 QC sample inserted between every 20 samples. Ionization signals were tain QC samples based on the intensities of internal standards for individual lipid classes to ensure no drop in intensity (within 20%) and no drift in retention time (within 0.05 min) throughout the run. Lipid levels were expressed in moles per L (mol/L) for plasma for statistical analyses.

Quality control (QC) samples were prepared using mixed plasma samples, with 1 QC sample inserted between every 20 tested samples. A total of 10 and 11 QC samples were inserted during the lipidomics profiling for plasma samples in the discovery and validation stage, respectively.

Ionization signals were monitored in QC samples based on the intensities of internal standards for individual lipid classes to ensure no significant drop in intensity (within 20%) and no drift in retention time (within 0.05 min) throughout the run. Lipids were identified based on structure-specific multiple reaction monitoring (MRMs), which comprise MRMs specific to both head groups distinct to individual lipid classes and fatty acyl compositions, as well as correct retention times by comparing to authentic lipid reference compounds from human lipid ID inventory constructed in-house. Lipid levels were expressed in moles per L (mol/L) of plasma for statistical analyses.

Supplementary figure legends

Figure S1 Quality control (QC) of the targeted lipidomics analysis

A. PCA analysis on the study and QC samples in the discovery stage;

B. PCA analysis on the study and QC samples in the validation stage;

C. Spearman's correlation coefficients of 10 QC runs in the discovery stage;

D. Spearman's correlation coefficients of 11 QC runs in the validation stage;

E. Intensity distribution of lipid species for study samples and QC runs in the discovery stage;

F. Intensity distribution of lipid species for study samples and QC runs in the validation stage.

CAG, chronic atrophic gastritis; GC, gastric cancer; IM, intestinal metaplasia; LGIN, low-grade intraepithelial neoplasia; PCA, principle component analysis; QC, quality control; SG, superficial gastritis.

Figure S2 Pearson correlation matrix for the validated lipids

Pearson correlation coefficients were calculated to represent pairwise correlation between each lipid. The magnitude of correlation coefficients varies by color, and only the significant correlation coefficients ($P < 0.05$) are presented in cells.

FFA, free fatty acid; LPI, lysophosphatidylinositol; LysoPC, lysophosphatidylcholine; PA, phosphatidic acid; PC, phosphatidylcholine.

Figure S3 Latent profiles revealing specific patterns of lipid alterations behind GC and gastric lesion progression

A. The lipids' latent profiles of the validation cohort. Ten latent vectors were obtained by VAEN. Each row represents a subject with different gastric histopathology, as s annotated in the right side. Each column represents a latent vector dimension. The relative value of a latent vector in research subjects is displayed in each cell with gradient color from blue (low) to red (high).

B. The selected latent vector dimensions over the 200 times running of the elastic net model.

Each row represents a running of the model and each column represents a latent vector dimension. For each latent dimension, the total times of being selected among the 200 runs are shown above. The R^2 in multivariate linear regression for each run is displayed in the right bar-plot. Black (not selected) or white-colored (selected) cells indicate the selection result of each latent dimensions during a specific run.

CAG, chronic atrophic gastritis; EN, elastic net; GC, gastric cancer; HGIN, high-grade intraepithelial neoplasia; IM, intestinal metaplasia; LGIN, low-grade intraepithelial neoplasia; SG, superficial gastritis; VAEN, variational auto-encoder followed by the elastic net regression model.

Figure S4 Pathway analysis on the proteins significantly associated with the risk of GC

Proteins significantly associated with the risk of GC occurrence were included for pathway analysis. P-values for each enriched pathway are shown in the bar-plot, with the table below indicating the proteins involved in each pathway.

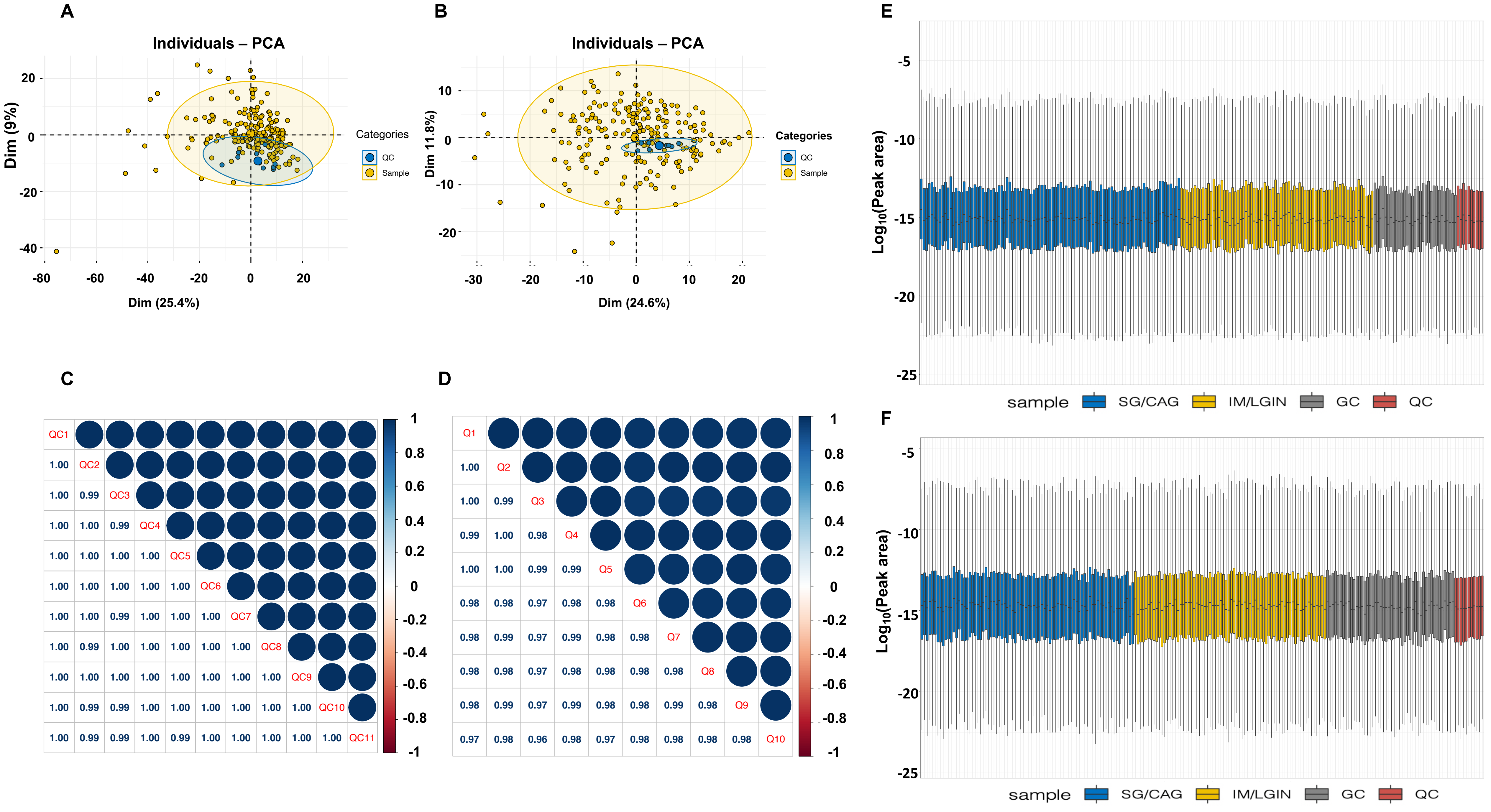
A
Individuals – PCA
Dim (9%)
Dim (25.4%)
Categories
QC
Sample
B
Individuals – PCA
Dim 11.8%)
Dim (24.6%)
Categories
QC
Sample
C
QC1
1.00 QC2
1.00 0.99 QC3
1.00 1.00 0.99 QC4
1.00 1.00 1.00 1.00 QC5
1.00 1.00 1.00 1.00 1.00 QC6
1.00 1.00 0.99 1.00 1.00 1.00 QC7
1.00 0.99 1.00 1.00 1.00 1.00 1.00 QC8
1.00 1.00 1.00 1.00 1.00 1.00 1.00 1.00 QC9
1.00 0.99 0.99 1.00 1.00 1.00 1.00 1.00 1.00 QC10
1.00 0.99 0.99 1.00 0.99 1.00 1.00 1.00 1.00 1.00 QC11
D
Q1
1.00 Q2
1.00 0.99 Q3
0.99 1.00 0.98 Q4
1.00 1.00 0.99 0.99 Q5
0.98 0.98 0.97 0.98 0.98 Q6
0.98 0.99 0.97 0.99 0.98 0.98 Q7
0.98 0.98 0.97 0.98 0.98 0.98 0.98 Q8
0.98 0.99 0.97 0.99 0.98 0.98 0.99 0.98 Q9
0.97 0.98 0.96 0.98 0.97 0.98 0.98 0.98 0.98 Q10
E
Log10(Peak area)
sample SG/CAG IM/LGIN GC QC
F
Log10(Peak area)
sample SG/CAG IM/LGIN GC QC

Figure S1

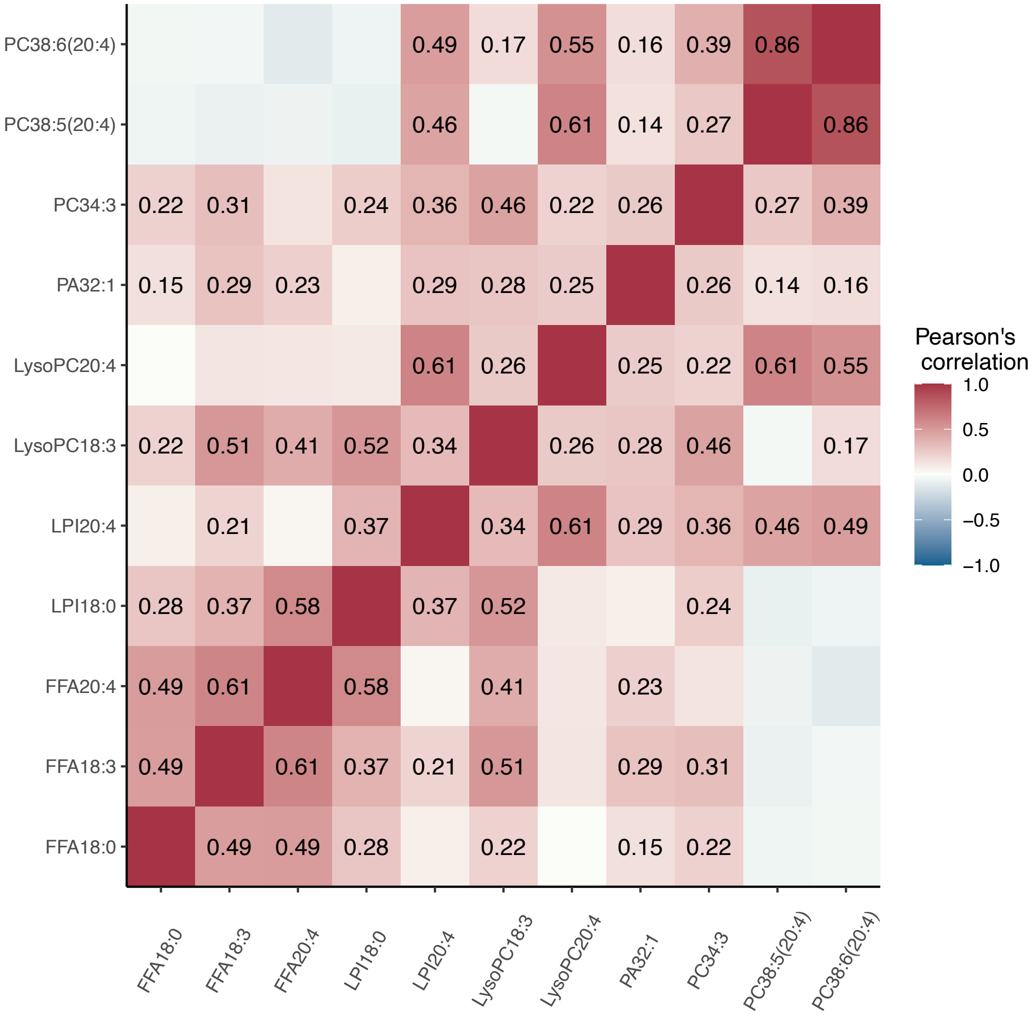
PC38:6(20:4)
0.49 0.17 0.55 0.16 0.39 0.86
PC38:5(20:4)
0.46 0.61 0.14 0.27 0.86
PC34:3
0.22 0.31 0.24 0.36 0.46 0.22 0.26 0.27 0.39
PA32:1
0.15 0.29 0.23 0.29 0.28 0.25 0.26 0.14 0.16
LysoPC20:4
0.61 0.26 0.25 0.22 0.61 0.55
LysoPC18:3
0.22 0.51 0.41 0.52 0.34 0.26 0.28 0.46 0.17
LPI20:4
0.21 0.37 0.34 0.61 0.29 0.36 0.46 0.49
LPI18:0
0.28 0.37 0.58 0.37 0.52 0.24
FFA20:4
0.49 0.61 0.58 0.41 0.23
FFA18:3
0.49 0.61 0.37 0.21 0.51 0.29 0.31
FFA18:0
0.49 0.49 0.28 0.22 0.15 0.22
FFA18:0
FFA18:3
FFA20:4
LPI18:0
LPI20:4
LysoPC18:3
LysoPC20:4
PA32:1
PC34:3
PC38:5(20:4)
PC38:6(20:4)
Pearson's correlation
1.0
0.5
0.0
−0.5
−1.0

Figure S2

A

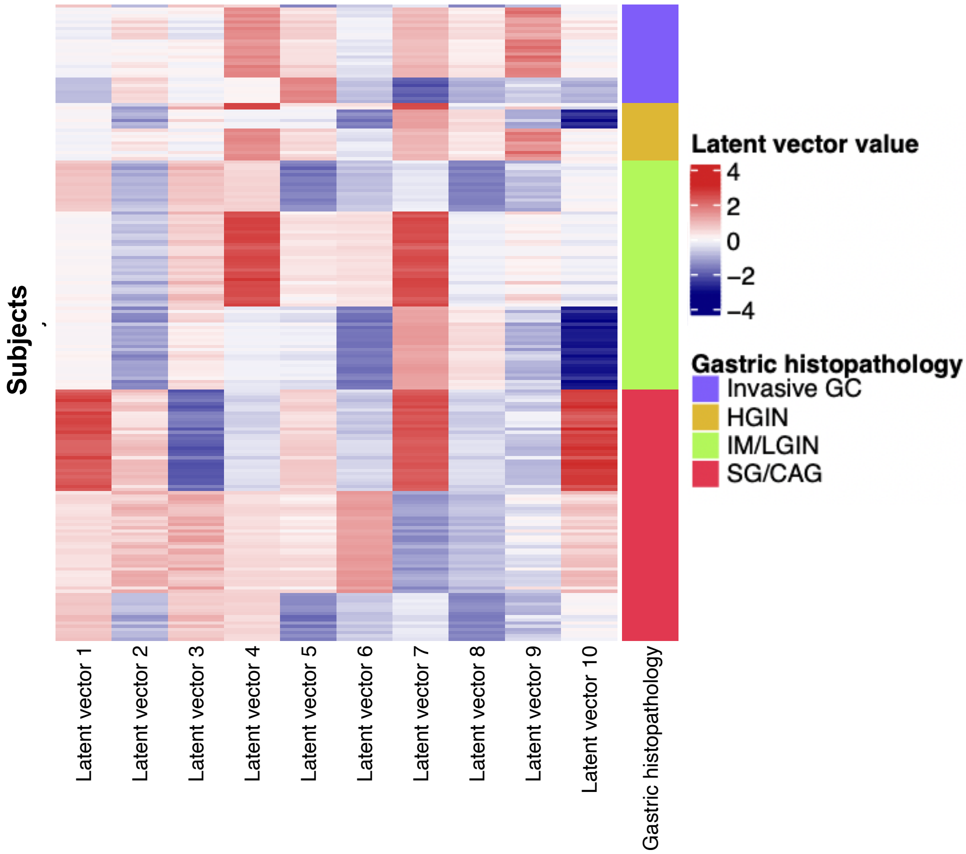

Subjects
Latent vector value
4
2
0
−2
−4
Gastric histopathology
Invasive GC
HGIN
IM/LGIN
SG/CAG
Latent vector 1
Latent vector 2
Latent vector 3
Latent vector 4
Latent vector 5
Latent vector 6
Latent vector 7
Latent vector 8
Latent vector 9
Latent vector 10
Gastric histopathology

B

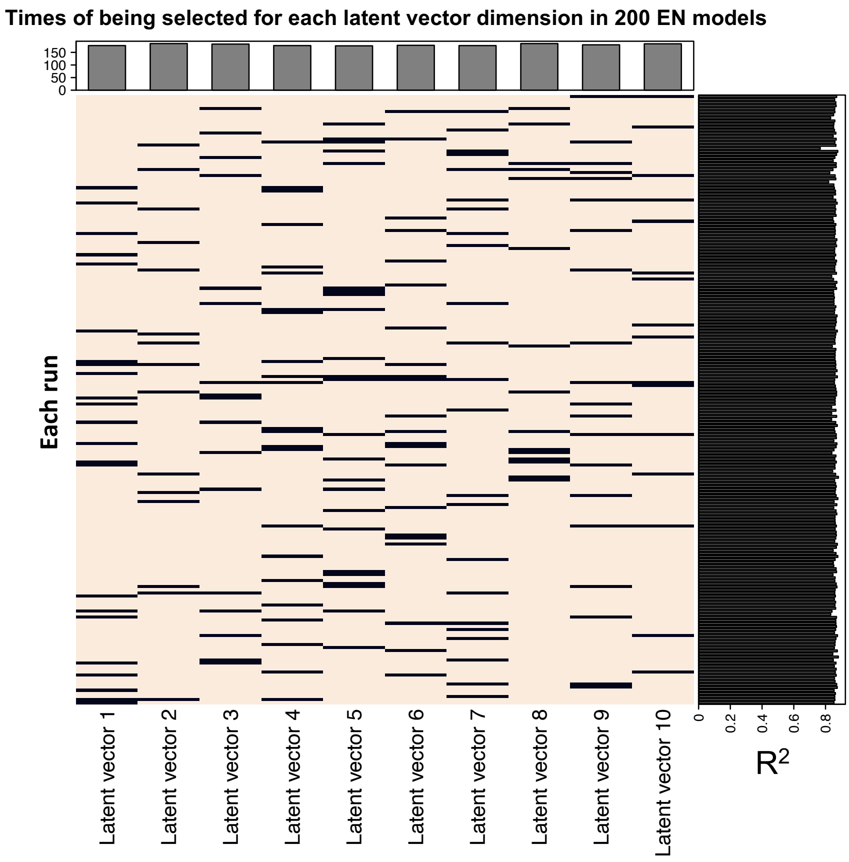

Times of being selected for each latent vector dimension in 200 EN models
150
100
50
0
Each run
Latent vector 1
Latent vector 2
Latent vector 3
Latent vector 4
Latent vector 5
Latent vector 6
Latent vector 7
Latent vector 8
Latent vector 9
Latent vector 10
0
0.2
0.4
0.6
0.8
R2

Figure S3

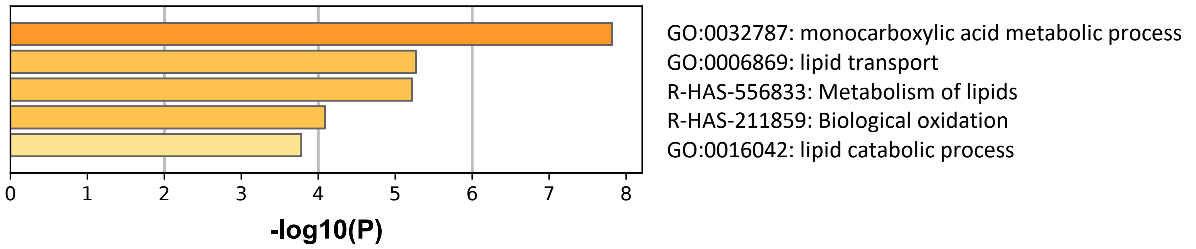

Pathway and Process	Involved proteins
GO:0032787: Monocarboxylic acid metabolic process	ASAH1, ATP8B1, CES2, LYPLA2, PTGS1, SLC27A3
GO:0006869: Lipid transport	ATP8B1, PITPNA, PITPNB, SLC27A3
R-HAS-556833: Metabolism of lipids	ASAH1, BDH1, PITPNB, PTGS1, SLC27A3
R-HAS-211859: Biological oxidations	ACY1, CES2, PTGS1
GO:0016042: Lipid catabolic process	ASAH1, LYPLA2, PAFAH1B

Figure S4

Table S1 Characteristics of the subjects in each study cohort

	Discovery cohort (*n*=200)	Validation cohort (*n*=200)				
	Total	Total	Prospective cohort (*n*=152)	Multi-time point longitudinal sub-cohort (*n*=76)	*P* value[a]	*P* value[b]
**Age (mean±SD)**	56.8±7.53	57.5±8.11	56.6±8.31	56.8±8.27	.606	0.334
**Sex**						
Male	124 (62.0%)	64 (32.0%)	57 (37.5%)	26 (34.2%)	.56	<0.001
Female	76 (38.0%)	136 (68.0%)	95 (62.5%)	50 (65.8%)		
***H.pylori* infection**						
No	102 (51.0%)	93 (46.5%)	73 (48.0%)	38 (50%)	.868	0.424
Yes	98 (49.0%)	107 (53.5%)	79 (52.0%)	38 (50%)		
**Gastric Histopathology**						
GC	31 (15.5%)	48 (24.0%)	-	-		
IM/LGIN	72 (36.0%)	73 (36.5%)	72 (47.4%)	33 (43.4%)	.795[c]	0.064
SG/CAG	97 (48.5%)	79 (39.5%)	80 (52.6%)	43 (56.6%)		

CAG, chronic atrophic gastritis; GC, gastric cancer including high-grade intraepithelial neoplasia and invasive gastric cancer; *H. pylori, Helicobacter pylori*; IM, intestinal metaplasia; LGIN, low-grade intraepithelial neoplasia; SG, superficial gastritis.

[a] The Kruskal-Wallis test or Pearson's χ^2 test was conducted for comparison of the validation cohort, prospective cohort, and multi-time point longitudinal sub-cohort.

[b] The Wilcoxon's rank sum test or Pearson's χ^2 test was conducted for comparison of the discovery and validation cohort.

[c] Analyses were only conducted for the categories of SG/CAG and IM/LGIN because GC cases were not prospectively followed.

Table S2 Lipids associated with the risk of GC shown in the untargeted metabolomics and targeted lipidomics analysis[a]

Lipid[b]	Discovery stage				Validation stage				Follow-up			
	GC vs SG/CAG				GC vs SG/CAG				Progression vs Non-progression			
	Untargeted metabolomics		Targeted lipidomics		Untargeted metabolomics		Targeted lipidomics		Untargeted metabolomics		Targeted lipidomics	
	OR	*P* value	OR	*P* value	OR	*P* value	OR	*P* value	OR	*P* value	OR	*P* value
α-Linolenic acid (FFA18:3)	0.37	3.87×10^{-4}	0.37	6.68×10^{-4}	0.53	0.003	0.56	0.004	0.64	0.020	0.69	0.025
Arachidonic acid (FFA20:4)	0.51	0.015	0.57	.017	0.70	0.046	0.71	0.038	0.90	0.300	0.73	0.022
sn-1 LysoPC18:3	0.27	1.11×10^{-4}	0.23	3.72×10^{-5}	0.62	0.010	0.81	0.139	0.82	0.170	0.79	0.049
Linoleic acid (FFA18:2)	0.43	0.002	0.68	0.141	0.56	0.010	0.63	0.018	0.69	0.045	0.75	0.050
Palmitic acid (FFA16:0)	0.47	0.007	0.69	0.068	0.56	0.010	0.61	0.014	0.63	0.020	0.78	0.057
sn-2 LysoPC20:3	0.29	1.04×10^{-4}	0.80	0.265	0.65	0.030	1.02	0.459	1.13	0.270	1.26	0.454

[a] The results shown here for untargeted metabolomics were extracted from our recent published study [4], while the results for targeted lipidomics are for the current study.

[b] These lipids had the most robust association with risk of GC in our recent published untargeted metabolomics study [4].

Table S3 Pathway analysis on the validated lipids

Pathway name	*P* value[a]	FDR-q	Pathway impact[b]	Match status[c]	Involved lipids
α-Linolenic acid metabolism	6.41×10^{-4}	0.003	0.25	2/13	Phosphatidylcholines; FFA18:3
Glycerophospholipid metabolism	0.005	0.015	0.12	2/36	Phosphatidylcholines; LysoPC20:4
Arachidonic acid metabolism	0.005	0.015	0.36	2/36	Phosphatidylcholines; FFA20:4
Linoleic acid metabolism	0.016	0.016	0.25	1/5	Phosphatidylcholines

[a] *P* value was calculated based on the Fisher's exact test.

[b] Pathway impact is a combination of the centrality and pathway enrichment results. It was calculated adding up the importance measures of each of the matched metabolites and then dividing by the sum of the importance measures of all metabolites in each pathway.

[c] The number of matched lipids for each pathway is presented here.

Table S4 The matched proteins associated with the validated lipids

Gene Symbol	GC vs SG&CAG[a]		GC vs IM&LGIN[a]		Biologically related lipids[b]
	OR	*P* value	OR	*P* value	
PEBP1	0.22	1.18×10^{-5}	1.05	0.848	PC38:6, PC34:3
LYPLA2	0.36	3.00×10^{-4}	0.97	0.880	PC38:6, PC34:3, LPC18:3, LPC20:4
PTGS1	2.65	0.001	0.93	0.697	FFA20:4
PITPNB	2.08	0.003	1.02	0.913	PC38:6, PC34:3
PAFAH1B2	0.56	0.007	1.12	0.650	LPC18:3, LPC20:4
ATP8B1	0.30	0.009	1.04	0.854	PC38:6, PC38:5, PC34:3
PITPNA	2.36	0.013	0.87	0.496	PC38:6, PC34:3
ASAH1	0.70	0.028	1.31	0.267	FFA20:4, FFA18:0
SLC27A3	23.50	0.034	1.29	0.406	FFA20:4, FFA18:0
BDH1	2.46	0.042	1.04	0.854	PC38:6, PC34:3
CYP2S1	0.67	0.057	1.34	0.236	FFA20:4
LYPLA1	0.22	0.069	0.93	0.726	PC38:6, PC34:3, LPC18:3, LPC20:4
ARFGAP1	1.56	0.128	0.89	0.558	PC38:6, PC34:3
CES1	1.99	0.192	2.07	0.227	FFA20:4, FFA18:0
CES2	0.70	0.194	0.66	0.042	FFA20:4, FFA18:0
LPCAT3	0.85	0.394	1.19	0.477	PC38:6, PC38:5, PC34:3, LPC18:3, LPC20:4
ACOT7	1.18	0.463	0.86	0.504	FFA20:4, FFA18:3, FFA18:0
LIPF	0.90	0.525	1.72	0.356	FFA20:4, FFA18:0
CLC	0.92	0.623	1.00	0.998	PC38:6, PC34:3, LPC18:3, LPC20:4, FFA18:0
FASN	0.92	0.673	0.79	0.24	FFA20:4, FFA18:0
PCYT1A	1.06	0.767	0.90	0.635	PC38:6, PC34:3
ACY1	0.95	0.864	0.61	0.019	FFA20:4, FFA18:0
PAFAH1B3	0.98	0.921	0.97	0.876	LPC18:3, LPC20:4

[a] The results shown here were extracted from our recent published proteomics study [5]

[b] The lipids are biologically related to the proteins based on the mapping from biological pathways annotated by the Human Metabolite Database.

Reference

1. Lu J, Lam SM, Wan Q, Shi L, Huo Y, Chen L, et al. High-coverage targeted lipidomics reveals novel serum lipid predictors and lipid pathway dysregulation antecedent to type 2 diabetes onset in normoglycemic chinese adults. Diabetes Care. 2019; 42: 2117–26.

2. Shui G, Guan XL, Low CP, Chua GH, Goh JSY, Yang H, et al. Toward one step analysis of cellular lipidomes using liquid chromatography coupled with mass spectrometry: application to saccharomyces cerevisiae and schizosaccharomyces pombe lipidomics. Mol Biosyst. 2010; 6: 1008.

3. Shui G, Cheong WF, Jappar IA, Hoi A, Xue Y, Fernandis AZ, et al. Derivatization-independent cholesterol analysis in crude lipid extracts by liquid chromatography/mass spectrometry: applications to a rabbit model for atherosclerosis. J Chromatogr A. 2011; 1218: 4357–65.

4. Huang S, Guo Y, Li ZW, Shui G, Tian H, Li BW, et al. Identification and validation of plasma metabolomic signatures in precancerous gastric lesions that progress to cancer. JAMA Netw Open. 2021; 4: e2114186.

5. Li X, Zheng NR, Wang LH, Li ZW, Liu ZC, Fan H, et al. Proteomic profiling identifies signatures associated with progression of precancerous gastric lesions and risk of early gastric cancer. EBioMedicine. 2021; 74: 103714.
